# Presence of a biofilm beneficiary alters the evolutionary trajectory of a biofilm former

**DOI:** 10.1093/ismejo/wraf160

**Published:** 2025-07-31

**Authors:** Xinli Sun, Zhihui Xu, Guohai Hu, Jiyu Xie, Yun Li, Lili Tao, Nan Zhang, Weibing Xun, Youzhi Miao, Ruifu Zhang, Qirong Shen, Christian Kost, Ákos T Kovács

**Affiliations:** Jiangsu Provincial Key Lab for Solid Organic Waste Utilization, Key lab of organic-based fertilizers of China, Jiangsu Collaborative Innovation Center for Solid Organic Wastes, Educational Ministry Engineering Center of Resource-saving fertilizers, Nanjing Agricultural University, Nanjing 210095, Jiangsu Province, China; Bacterial Interactions and Evolution Group, DTU Bioengineering, Technical University of Denmark, Kongens Lyngby 2800, Denmark; Jiangsu Provincial Key Lab for Solid Organic Waste Utilization, Key lab of organic-based fertilizers of China, Jiangsu Collaborative Innovation Center for Solid Organic Wastes, Educational Ministry Engineering Center of Resource-saving fertilizers, Nanjing Agricultural University, Nanjing 210095, Jiangsu Province, China; Bacterial Interactions and Evolution Group, DTU Bioengineering, Technical University of Denmark, Kongens Lyngby 2800, Denmark; China National GeneBank, BGI Research, Shenzhen 518120, Guangdong Province, China; BGI Research, Shenzhen 518083, Guangdong Province, China; Shenzhen Key Laboratory of Environmental Microbial Genomics and Application, BGI Research, Shenzhen 518083, Guangdong Province, China; Jiangsu Provincial Key Lab for Solid Organic Waste Utilization, Key lab of organic-based fertilizers of China, Jiangsu Collaborative Innovation Center for Solid Organic Wastes, Educational Ministry Engineering Center of Resource-saving fertilizers, Nanjing Agricultural University, Nanjing 210095, Jiangsu Province, China; Institute of Biology, Leiden University, Leiden 2333 BE, The Netherlands; Jiangsu Provincial Key Lab for Solid Organic Waste Utilization, Key lab of organic-based fertilizers of China, Jiangsu Collaborative Innovation Center for Solid Organic Wastes, Educational Ministry Engineering Center of Resource-saving fertilizers, Nanjing Agricultural University, Nanjing 210095, Jiangsu Province, China; Jiangsu Provincial Key Lab for Solid Organic Waste Utilization, Key lab of organic-based fertilizers of China, Jiangsu Collaborative Innovation Center for Solid Organic Wastes, Educational Ministry Engineering Center of Resource-saving fertilizers, Nanjing Agricultural University, Nanjing 210095, Jiangsu Province, China; Jiangsu Provincial Key Lab for Solid Organic Waste Utilization, Key lab of organic-based fertilizers of China, Jiangsu Collaborative Innovation Center for Solid Organic Wastes, Educational Ministry Engineering Center of Resource-saving fertilizers, Nanjing Agricultural University, Nanjing 210095, Jiangsu Province, China; Jiangsu Provincial Key Lab for Solid Organic Waste Utilization, Key lab of organic-based fertilizers of China, Jiangsu Collaborative Innovation Center for Solid Organic Wastes, Educational Ministry Engineering Center of Resource-saving fertilizers, Nanjing Agricultural University, Nanjing 210095, Jiangsu Province, China; Jiangsu Provincial Key Lab for Solid Organic Waste Utilization, Key lab of organic-based fertilizers of China, Jiangsu Collaborative Innovation Center for Solid Organic Wastes, Educational Ministry Engineering Center of Resource-saving fertilizers, Nanjing Agricultural University, Nanjing 210095, Jiangsu Province, China; Jiangsu Provincial Key Lab for Solid Organic Waste Utilization, Key lab of organic-based fertilizers of China, Jiangsu Collaborative Innovation Center for Solid Organic Wastes, Educational Ministry Engineering Center of Resource-saving fertilizers, Nanjing Agricultural University, Nanjing 210095, Jiangsu Province, China; Jiangsu Provincial Key Lab for Solid Organic Waste Utilization, Key lab of organic-based fertilizers of China, Jiangsu Collaborative Innovation Center for Solid Organic Wastes, Educational Ministry Engineering Center of Resource-saving fertilizers, Nanjing Agricultural University, Nanjing 210095, Jiangsu Province, China; Department of Ecology, School of Biology/Chemistry, University of Osnabrück, Osnabrück 49076, Germany; Bacterial Interactions and Evolution Group, DTU Bioengineering, Technical University of Denmark, Kongens Lyngby 2800, Denmark; Institute of Biology, Leiden University, Leiden 2333 BE, The Netherlands

**Keywords:** bacterial interaction, biofilm, experimental evolution, genomics, fitness, Bacillus velezensis, Stutzerimonas degradans

## Abstract

Biofilm evolution is typically studied in monocultures or in communities displaying mutualistic or exploitative interactions. However, in communities with fluctuating interactions, the influence of biofilm-beneficiary bacteria on the evolution of biofilm-founder bacteria remains less understood. Biofilm-beneficiary bacteria cannot form robust biofilms independently but can incorporate into the biofilm of biofilm-formers, thereby gaining the ability to colonize given niche. In this study, we demonstrate that the biofilm-former *Bacillus velezensis* SQR9 reproducibly diversified into biofilm-enhanced slimy and biofilm-weakened rough types, both in the presence and absence of a biofilm-beneficiary *Stutzerimonas degradans* XL272 (formerly *Pseudomonas stutzeri*), but with variable frequencies under the two conditions. The exopolysaccharide producer slimy types dominated *B. velezensis* populations in monoevolution, whereas the exploiter rough types, which exploit the exopolysaccharides produced by the slimy types, dominate in coculture evolution. Phenotypic changes in *B. velezensis* were linked to mutations in specific genes that regulate biofilm formation and sporulation, including *ywcC*, *comA*, *comP*, *degS*, *degQ*, and *spo0F*. A frameshift mutation in the *cpsA* gene of *S. degradans* increased its exopolysaccharide production in the dual-species biofilm, which served as shared resources and allow the *B. velezensis* exploiter (i.e. rough type) to outcompete the producer (i.e. slimy type) during coculture evolution. Additionally, longitudinal population sequencing and “replay” evolution experiments with the *S. degradans* mutant revealed that the *cpsA* mutation accelerated the fixation of the rough type within *B. velezensis* populations. In conclusion, this research demonstrates that interspecific interactions can adaptively favor exploiters within biofilm populations.

## Introduction

Biofilms are complex communities formed by microbial cells that aggregate and adhere to biological or abiotic surfaces, creating multi-layered structures [1]. Biofilms are prevalent in natural environments, such as rivers, oceans, and soils, as well as in engineered settings like water treatment facilities and pipeline systems [2]. Biofilms mainly consist of microbial cells and extracellular matrix, which consists of various macromolecules, including polysaccharides, proteins, nucleic acids, and lipids [3]. The extracellular matrix forms a complex network that provides structural support and protection against environmental stress [3, 4]. As a shared resource among neighboring cells, it also represents a typical example of microbial public goods—costly to produce but susceptible to exploitation [2].

Interactions among cells within biofilms can drive evolutionary change. In mono-species biofilms, different ecotypes evolve that occupy overlapping niches and generate facilitative, exploitative, or competitive interactions [5]. Evolutionary outcomes that are commonly observed in the evolution of mono-species biofilm include: (i) the emergence of mutants with niche specificity and character displacement [6–9]; (ii) the evolution of mutants that increase polysaccharides secretion, along with other types that produce none or less [10–12]; or (iii) division of labor in extracellular matrix production [13]. Key factors driving this kind of adaptive diversification are, e.g. intraspecific competition [14], spatially heterogeneous environment [15], eco-evolutionary feedbacks [16], trade-offs [17], and clonal interference [18]. However, the corresponding evolutionary outcomes can change if environmental conditions fluctuate [19]. For instance, non-producers of a certain metabolite can evolve to become producers [20], and division of labor in the extracellular matrix can break down and evolve to autonomy [21].

In contrast to mono-species biofilms, multi-species biofilms involve a more intricate web of interactions, as diverse microbial species coexist and interact within the same physical space. Interspecies interactions can not only drive evolutionary change but also stabilize biofilm-enhancing variants, thus promoting mutualistic interactions and enhancing biofilm formation [22–24]. The evolutionary dynamics within multi-species biofilms can be strongly influenced by factors such as the type and availability of nutrient sources and environmental fluctuations [25, 26]. In general, interspecies interactions are thought to drive diversification by introducing new selective pressures and ecological opportunities [24, 27, 28]. However, in certain scenarios, the evolution of intraspecific diversity may be constrained in multi-species biofilm, particularly when certain functions are already fulfilled by a partner species [29]. Another factor that has been identified to drive the evolution and maintenance of phenotypic diversification in biofilms is spatial heterogeneity of environmental factors [8, 24]. This kind of heterogeneity allows phenotypically different strains to coexist, thus reducing competition for shared resources. Overall, the study of biofilm evolution provides valuable insights into the emergence and maintenance of microbial diversity.

In previous research, a dual-species biofilm consortium was established consisting of *Bacillus velezensis* SQR9 and *Stutzerimonas degradans* XL272 [30]. *S. degradans* XL272 is unable to form a structured pellicle—a biofilm at the air-liquid interface—when grown in isolation, producing only a weak or incomplete biofilm. However, it can incorporate into the robust pellicles formed by *B. velezensis* SQR9, establishing a producer-beneficiary relationship. This system provides a valuable model to investigate how the presence of a beneficiary affects the evolutionary trajectory of a biofilm-forming producer. To address this, we studied the evolution of the biofilm former *B. velezensis* under monoculture conditions and compared it to its evolution in coculture with the beneficiary *S. degradans*. Based on previous studies that analysed the experimental evolution of biofilms, we anticipated the emergence of phenotypically distinct *B. velezensis* variants differing in biofilm formation and exopolysaccharide production as a key public good. We also hypothesized that coculture evolution within biofilms would intensify the dependence of the beneficiary *S. degradans* on the biofilm former *B. velezensis*. These hypotheses were tested by comparing the phenotypic and genotypic differences between ancestors and evolved genotypes under both monoculture and coculture conditions. Finally, to test whether specific phenotypic changes observed in coculture evolution were causally linked to genetic mutations, we conducted a “replay” coculture evolution experiment using a *S. degradans* mutant.

## Materials and methods

### Strains and media

*B. velezensis* strain SQR9 and *S. degradans* strain XL272 were the ancestral strains used in the evolution experiments. *B. velezensis* SQR9 was transformed with a plasmid containing a GFP marker, *S. degradans* XL272 was modified with a mini-Tn7 transposon containing a dsRed marker. Tryptone soya broth (TSB) (Sigma-Aldrich, CAT# 22098) was used for all experimental evolution and phenotypic characterization. Lysogeny broth (LB) was used during genetic engineering. Solid media contained 1.5% agar added to TSB or LB media. All strains were stored at −80°C using overnight grown cultures in TSB or LB media supplemented with 25% glycerol before freezing the samples.

Start inoculum across the study was obtained by growing the cells overnight to exponential phase in TSB medium at 30°C, 180 rpm shaken condition. The cells were spun down and diluted to OD_600_ of 1 in 0.9% NaCl buffer. For coculture, the start inoculum was prepared by mixing equal volumes of isolates.

### Experimental evolution

Approximate 10^6^ cells of *B. velezensis* or a mixture of *B. velezensis* and *S. degradans* were inoculated in non-adjacent wells of two independent 24-well cell culture plates (VWR, CAT# 10062–898) and incubated at 30°C in TSB supplemented with 5 mg/l chloramphenicol without shaking. Addition of chloramphenicol was to preserve the plasmid in *B. velezensis* and to avoid contamination. Because *S. degradans* cannot form robust biofilm independently, monoevolution of *S. degradans* was not performed in this study. Every 2 days, mature biofilms from the first plate were harvested with sterile inoculation loop, disrupted and re-inoculated in two independent plates through a 1:100 dilution (20 μl in 2 ml of fresh TSB + chloramphenicol medium) according to the method described in literature [10]. The remaining populations were mixed with 1 ml of 50% glycerol solution and preserved as frozen stocks (−80°C). The second plate was imaged using an Axio Zoom V16 stereomicroscope (Carl Zeiss, Jena, Germany) equipped with a Zeiss CL 9000 LED light source and an AxioCam MRm monochrome camera (Carl Zeiss) equipped with HE eGFP (excitation at 470/40 nm and emission at 525/50 nm) and HE mRFP (excitation at 572/25 nm and emission at 629/62 nm) filter sets. After the 1^st^, 3^rd^, 6^th^, 9^th^, 12^th^, 15^th^, and 18^th^ transfer, 10% of the disrupted populations were sonicated (24^*^1 s pulses at 20% amplitude with 1 s pause between the pulses), serial diluted and plated on TSB plates supplemented with either 5% NaCl for *B. velezensis* or 20 mg/l gentamicin for *S. degradans*. Colonies with divergent morphotypes were randomly selected and preserved at −80°C after overnight cultivation in TSB medium and supplementation of the culture with 25% glycerol.

### Phenotypic characterizations

(1) Colony morphology assay. Colony morphologies were examined on TSB medium with 1.5% agar. The plates were dried under laminar airflow conditions for 40 minutes after solidifying. Glycerol stocks were streaked out and incubated at 30°C until colonies were visible. Single colonies were re-streaked out on fresh plates and incubated at 30°C. Photos were taken at 2–3 days for *B. velezensis* and 6 days for *S. degradans*.

(2) Biofilm morphology assay. 20 μl of the start inoculum was cultivated in 2 ml of TSB medium in a 24-well microplate (Fisher Scientific). The microplates were incubated statically at 30°C for 48 h.

(3) Spore observation and quantification. Biofilms formed after 2 and 7 days were collected and resuspended in 2 ml of PBS buffer with glass beads, then vortexed vigorously for 5 minutes to disrupt the pellicles into single cells and aggregates. These disrupted cells were observed using Phase Contrast and Darkfield Microscopy (Olympus CX41). Spores appear transparent, whereas vegetative cells and dead cells appear black. 1 ml of the disrupted suspension was heat-treated at 70°C for 15 minutes to kill vegetative cells. Both the disrupted and heat-treated samples underwent sonication (30% amplitude, 30 cycles of 1 s sonication with 1 s rest) to further disaggregate cells. The total cell numbers and spore numbers were quantified using serial dilution plating and colony counting after incubation.

(4) Quantification of exopolysaccharide in the biofilm matrix. Biofilms were grown in 24-well microplates as described before. The supernatant was removed by syringe, biofilms were suspended in 2 ml water. Cells were separated from biofilm matrix by vortexing vigorously and centrifugation (10 000 g, 2 min), and the supernatant was filtered through 0.22 μm pore size filter. Total polysaccharides in the filtrate were quantified by the phenol-sulfuric acid assay with glucose as standard [31].

### Biofilm complementation assay

The rough morphotypes (BS1R, BS6R), which represent the matrix-exploiting variants of *B. velezensis*, were supplemented with the biofilm matrix or supernatant of the other isolates. The matrix donor strains contained *B. velezensis* ancestor, two coevolved slimy isolates (BS1S, BS4S), *S. degradans* ancestor (SA), the evolved variant (SE), and the mutant ∆*cpsA* (SM). 100 μl of the donor strains (OD_600_ ~ 1) were inoculated in 6-well microplates containing 10 ml TSB medium, and cultivated at 30°C for 48 h. The underneath supernatant was removed by 10 ml sterile syringe, the biofilms were resuspended in 10 ml water and vortexed rigorously for 15 mins. For *S. degradans*, the supernatant was collected instead. The collected biofilms were centrifuged at 10 000 g for 5 minutes, and filtered through 0.22 μm pore size filter. The biofilm matrix was mixed with equal volume of 2^*^TSB medium. Sterile water was mixed with 2^*^TSB as a control medium. 20 μl of the recipient strain was inoculated in these media in 24-well microplates and incubated statically at 30°C for 48 h. The supernatant was removed by sterile syringe, the biofilms were resuspended in 2 ml water and vortexed rigorously for 15 minutes. The biofilm suspension was transferred to 48-well plates and the biomass was determined by measuring the optical density at 600 nm. Each treatment contained six replicates.

### Construction of *B. velezensis* gene knockout mutant

Knockout mutants for the *ywcC* and *spo0F* genes were generated through DNA recombination with pheS^*^-*spc*, followed by counter selection [32]. Four fragments were amplified using corresponding primers and fused together by overlap PCR: the upstream fragments of the target gene, the downstream fragments of the target gene, the pheS^*^-*spc* fragments, and the target gene fragments. The fused fragments were introduced to the genome of *B. velezensis* by chemical transformation. Transformants were selected on LB plates containing spectinomycin. The spectinomycin-resistant transformants were then plated on LB plates containing 5 mM p-chloro-phenylalanine. Transformants carrying the pheS^*^ gene were eliminated, whereas the knockout mutants showed resistance to p-chloro-phenylalanine. Knockout mutants were validated by PCR and sequencing. The oligonucleotide primers used in this study are listed in Supplementary Dataset S1. Other knockout mutants were created by previous laboratory members using the same method and stocked in lab freezer.

### Construction of *S. degradans* gene knockout mutant

Knockout mutant for the *cpsA* gene was generated by DNA recombination forced by I-SceI endonuclease followed by counter selection [33, 34]. Upstream and downstream fragments were amplified by appropriate primers with recognition sites of I-SceI, digested by restriction enzymes, assembled by ligase, and cloned into pSNW4 vector. The resulting plasmid was introduced into *S. degradans* by triparental mating using pRK600, generating the integrant strain. The helper plasmid pQURE which encode the I-SceI endonuclease was introduced into the integrant strain by electroporation to force the double DNA recombination. Knockout mutant was validated by PCR and sequencing. The helper plasmid was cured by omitting the inducer compound from the medium. The oligonucleotide primers used in this study are listed in Supplementary Dataset S1.

### Cell numbers quantification in biofilms

To monitor population size throughout evolution and compare the biofilm biomass of evolved isolates, the cell numbers of both species in pellicles were quantified by absolute qPCR using the primers described in our previous publication [30]. For tracking the cell number changes throughout evolution, 500 μl of population from 1^st^, 3^rd^, 6^th^, 9^th^, 12^th^, 15^th^, and 18^th^ transfer was taken out from the frozen stocks. Each population only contained one sample at each transfer. To compare the biofilm biomass of evolved isolates, samples were collected from biofilms grown in TSB medium for 48 hours in 24-well plates. Each treatment included three biological replicates. Genomic DNA was extracted with EURx Bacterial & Yeast Genomic DNA Kit (CAT# E3580). qPCR was performed with Agilent’s Real Time PCR instrument Mx3005P. Reaction components are as follow: 7.2 μl H_2_O, 10 μl 2× Luna Universal qPCR Master Mix (NEB, CAT# M3003), 0.4 μl 10 μM of each primer and 2 μl template DNA. The PCR programs were carried out under the following condition: 95°C for 1 minute, 40 cycles of 95°C for 5 s, 60°C for 45 s, followed by a standard dissociation curve segment. Each sample was run in triplicates.

### Evolved clone genome re-sequencing and analysis

Sixty-nine *B. velezensis* evolved clones and sixteen *S. degradans* evolved clones were selected for genome resequencing. Four ancestors of each species were also re-sequenced to exclude common mutations arose from historical lab domestication instead of this study. The strain list is included in Supplementary Dataset S2. Genomic DNA of selected strains were extracted using the EURx Bacterial & Yeast Genomic DNA Kit from overnight cultures. Paired-end fragment reads (2 × 150 bp) were generated using a NovaSeq 6000 instrument (Illumina, San Diego, CA, USA). Raw reads were analyzed by Cutadapt (V1.9.1) to remove adaptors, primer sequences, content of N bases more than 10%, and bases of quality lower than 20. Clean reads were mapped to the reference genomes (CP046538.1 and CP009679.1) using BWA (V0.7.17). Mapping results were processed by Picard (V1.119) to remove duplication. Variants were called using GATK (V3.8.1) software and annotated using Annovar (V21 Apr 2018). Data on mutation frequencies for each strain sequenced are provided in Supplementary Dataset S2. Mutations in were then filtered in R to remove mutations (i) with low frequency (<95%), (ii) synonymous single nucleotide polymorphisms (SNPs), (iii) intergenic mutations, (iv) identified in only one clone. Subsequently, mutations identified in the ancestral genomes were filtered. The resulting table was visualized using the R *pheatmap* package (v1.0.13).

### Population metagenome sequencing and analysis

Whole populations were sequenced at 1^st^, 3^rd^, 6^th^, 9^th^, 12^th^, 15^th^, and 18^th^ transfer. All sixteen populations (eight from monoevolution, and eight from coculture evolution) and ancestor strains were sequenced. Genomic DNA were extracted from 1 ml of frozen populations using the EURx Bacterial & Yeast Genomic DNA Kit. The sequencing libraries were prepared using MGIEasy Fast FS DNA Library Prep Set (MGI Tech). Paired-end fragment reads (150 bp × 2) were generated on a DNBSEQ-Tx sequencer (MGI Tech) following the manufacturer’s procedures. All population samples were sequenced with ultrahigh depth data to ensure >300 × depth coverage for each species in monoevolved and coculture-evolved conditions. Raw data were filtered using SOAPnuke [35] (version 1.5.6) to remove low quality reads: reads including more than 50% of bases with quality lower than 12, reads including more than 10% of unknown base “N”, and reads containing adaptor contamination. For the coculture-evolved populations, the clean data were mapped to the *B. velezensis* SQR9 genome (GenBank accession number CP006890.1) and *S. degradans* XL272 genome (GenBank accession number CP046538.1) using bowtie2 with the parameters used in breseq [36, 37] (version 0.35.7), and the clean data of the monoevolved populations were only mapped to the SQR9 genome. Then reads which mapped to SQR9 genome or XL272 genome were extracted from bam file using samtools [38] (version 1.12), respectively. To ensure the similar variants calling sensitivity, we normalized the mapped data to 300× depth using seqtk (version 1.3-r107-dirty) (https://github.com/lh3/seqtk) for each species in each population sample for mutation calling. Mutations were called using breseq (version 0.35.7) with the default parameters and a -p option for population samples. The resulting mutations were provided in Supplementary Dataset S3.

We then filtered the mutations that found in the ancestor strains and those that did not reached a cumulative frequency of 5% within a lineage. The resulting mutation list was subjected to analysis and visualization. The Jaccard similarity coefficient was calculated using the following formula: the number of mutated genes shared by both lineages divided by the total number of mutated genes in both lineages.

After calculating the Jaccard similarity coefficient, we excluded transient mutations detected at only a single transfer time. We then plotted the mutation accumulation over time using a line chart and illustrated the distribution of mutation types with a stacked bar plot. We conducted a linear mixed model (LLM) to evaluate how two different evolutionary conditions (B-co and B-mono) influence the mutation counts in SQR9, factoring in multiple time points (transfers) and lineage variability. The model was fitted using the lme4 package (v1.1–37) in R, and post hoc comparisons were performed with the emmeans package (v1.11.1) to assess group differences at each transfer point. NMDS based on a Bray–Curtis dissimilarity matrix was performed and plotted using the R vegan package (v2.7–1) to explore the differences in the composition of mutations. PERMANOVA was conducted to evaluate the effects of evolution treatment on the composition of mutations by using the R vegan package.

We then filtered out mutations that occurred in intergenic regions, and visualized the mutational dynamics using muller plots. Muller plots were generated using the Lolipop package [39] (version 0.6) (https://github.com/cdeitrick/lolipop) using default parameters. This package predicts genotypes and populations based on shared trajectories of mutations over time and test their probability of nonrandom genetic linkage. Successive evolution of genotypes, or nested linkage, is identified by a hierarchical clustering method. Then muller plots were manually colored by genotypes.

### “Replay” evolution experiment

Four “replay” evolution treatments were set: (i) Mono-evolution of the *B. velezensis* ancestor (BA), (ii) Coculture evolution of the *B. velezensis* SQR9 ancestor and the *S. degradans* ancestor (BASA), (iii) Coculture evolution of the *B. velezensis* SQR9 ancestor and the *S. degradans* evolved variant (BASE), and (iv) Coculture evolution of the *B. velezensis* SQR9 ancestor and the *S. degradans* ∆*cpsA* mutant (BASM). The procedure followed the same protocol as the above-described experimental evolution. However, the “replay” evolution was only conducted for 6^th^ transfers. After each transfer, biofilm populations or communities were vortexed for 5 minutes at maximum speed, sonicated using 24 pulses of 1 s at 20% amplitude, with a 1-s pause between pulses. The samples were then serially diluted and plated on TSB plates supplemented with 5% NaCl. The proportion of *B. velezensis* SQR9 morphotype variants was recorded. For the newly identified rough variants, the complete sequences of the *comA*, *comP*, *degS*, and *degQ* genes were obtained by Sanger sequencing and aligned to ancestral gene sequences.

## Results

### Biofilm beneficiary *S. degradans* affects diversification of the biofilm former *B. velezensis*

**Figure 1:**
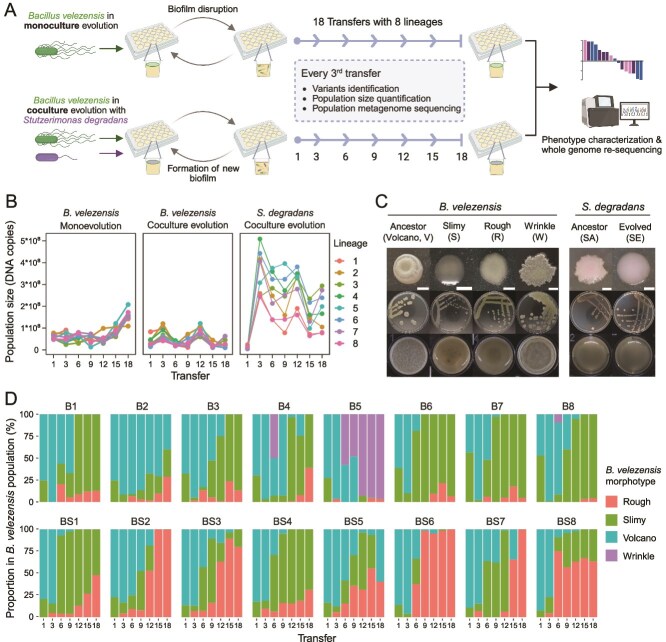
Population dynamics. (**A**) Workflow of biofilm evolution. The diagram was created on Biorender.com. (**B**) Dynamic changes in population size. The cell numbers were assessed every 3^rd^ transfer by qPCR. (**C**) Colony and pellicle phenotype of the evolved variants. The top row shows an enlarged view of a colony, the middle row shows the entire plate of streaked colonies, and the bottom row shows the pellicle phenotype. Colonies with different morphotypes were streaked on TSB agar plates and cultivated at 30°*C. petri* dish diameter is 9 cm, scale bar represents 0.2 cm, well diameter is 1.5 cm. Pellicles were cultivated in TSB liquid medium at 30°C, and photographs were taken 48-hour post-incubation. (**D**) the emergence and successional dynamics of *B. velezensis* evolved variants. “B” represents monoevolved populations, “BS” represents coculture-evolved communities.

To investigate how a biofilm beneficiary affects the evolution of a biofilm former, we performed an evolution experiment, in which the biofilm former *B. velezensis* SQR9 was either cultivated under monoculture conditions or in coculture with the beneficiary *S. degradans* (see Methods). Whereas *S. degradans* is unable to form robust biofilms, which leads to insufficient population size for serial transfer, monoculture evolution of this strain was not conducted. Biofilms were disrupted, diluted, and transferred to fresh medium every other day, for a total of 18 transfers (Fig. 1A). Each transfer corresponded to approximately six to seven generations. Pellicles were collected after 48 hours to provide sufficient time for biofilm maturation before transfer. By comparing their cell numbers in monoculture and cocultures, the interaction between *B. velezensis* and *S. degradans* changed from mutualism (+/+) to ammensalism (−/0) from 24 to 48 hours (Fig. S1).

At the onset of the evolution experiment, biofilms appeared dry, frosted, and fragile (Fig. S2). However, they became watery and glutinous at 6^th^ transfer and kept this morphology until the end of the experiment. In general, coculture-evolved biofilms were thicker and stickier than monoevolved biofilms. The dynamic changes of cell numbers in biofilm populations were assessed by qPCR. Over the course of the evolution experiment, cell numbers of all *B. velezensis* biofilm populations increased gradually under monoculture conditions, yet remained constant in cocultures (Fig. 1B). In all replicate cocultures, the size of *S. degradans* populations increased starkly until the 3^rd^ transfer and then declined towards the end of the experiment (Fig. 1B). In general, the overall cell number of coculture-evolved communities were significantly higher than that of the monoevolved populations (Welch’s t test, t(63.51) = 8.82, *P* < .001).

The colony morphologies of all strains were examined every 3^rd^ transfer on selective agar plates to identify variants (Fig. 1C) and monitor their successional dynamics (Fig. 1D). The ancestor of *B. velezensis* SQR9 is designated as volcano type. Three colony morphotypes were identified that evolved during the experiment: slimy, rough, and wrinkle types. The wrinkle morphotype only emerged in monoevolved populations and only persisted in one population (B5). The ancestor-like volcano morphotype was gradually replaced by the newly emerged variants and only persisted in a few of the terminal populations (BS5, B2). In *S. degradans* XL272, ancestral colonies showed an irregular colony edge, whereas evolved colonies were round. However, this difference is not discernible if the colonies are small, thus all evolved clones are referred to as “SE”.

Although *B. velezensis* showed similar morphotypes in mono- and cocultures, their successional dynamics were different: the proportion of rough morphotype within the evolving lineages was higher in coculture conditions than in monocultures (Welch’s t test, *P* < .001, t(60.98) = 5.73; Fig. 1D). During the final transfer, six of eight monoevolved populations contained a large proportion of slimy types and a small proportion of rough types. In coculture-evolved populations, however, the proportion of rough type greatly increased and completely took over in three out of eight populations. A possible explanation for the increased success of rough type in cocultures is the influence of *S. degradans*.

### Loss of sporulation in evolved *B. velezensis* isolates

We compared the phenotypic differences of the evolved clones, including spore formation and biofilm formation. Eight *B. velezensis* isolates (BS1-R, BS5-R, BS1-S, BS4-S, B2-R, B6-R, B1-S, B3-S) were selected, designated as CoR, CoS, MoR, and MoS, depending on their evolutionary conditions (coculture or monoculture evolved) and morphotypes (rough or slimy). Only the ancestor had the ability to form spores in biofilms at 2 and 7 days, whereas no spores were detectable in evolved isolates (Fig. S3). The sporulation rate of the ancestor was 2.0 ± 0.27% and 85 ± 6.4% for 2 and 7 days, respectively, whereas evolved isolates displayed a spore frequency below 0.1%, independent of mono- or co-culture evolution.

### B. velezensis rough types were disadvantaged in monoculture but favored in coculture with S. degradans

**Figure 2:**
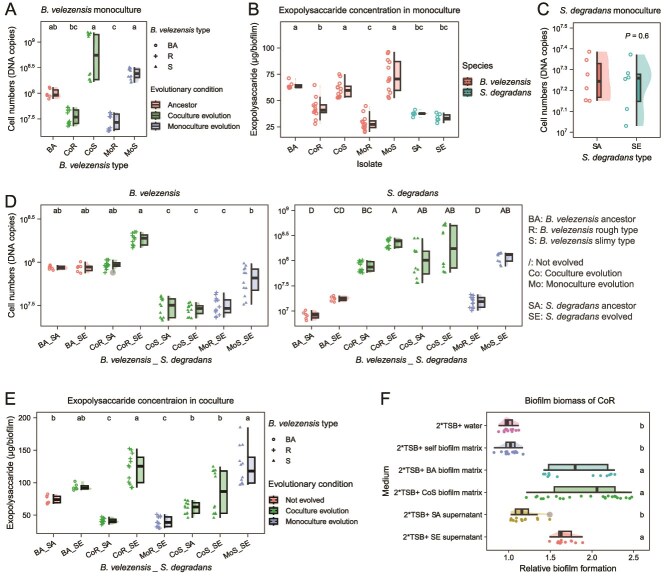
Comparison of ancestors and evolved isolates. (**A**) Cell numbers of *B. velezensis* in monocultured biofilms. “BA” (ancestor), “S” (slimy type), “R” (rough type), “Co” (coculture evolution), “Mo” (monoculture evolution); BA: six replicates, others: 12 replicates from two isolates. Statistical differences marked with different letters (*P* < .05) were assessed using Kruskal-Wallis test followed by Dunn's post hoc analysis with Benjamini-Hochberg correction unless stated otherwise. (**B**) Exopolysaccharide quantification in biofilms. “SA” (*S. degradans* ancestor), “SE” (*S. degradans* evolved isolate), each with six replicates. (**C**) Cell numbers of *S. degradans* in monocultured biofilms. Statistical difference between “SA” and “SE” was analyzed using t-test. (**D**) Cell numbers of *B. velezensis* and *S. degradans* in cocultured biofilms. Significant differences indicated as above. “BA_SA” and BA_SE”: six replicates, others: 12 replicates from two isolates. (**E**) Exopolysaccharide concentration in cocultured biofilms. Statistical significance marked with different letters (*P* < .05) were assessed using Kruskal-Wallis test, followed by Dunn's post hoc, no correction applied. (**F**) Relative biofilm of CoR isolates in medium supplemented with extracellular matrix of other isolates compared to that in TSB medium. Biofilm biomass was quantified by measuring the OD_600_ of vortexed biofilm. Biofilm formation of CoR isolates is enhanced by complementing them with the growth supernatant of evolved *S. degradans* (SE) and the extracellular matrix from the *B. velezensis* ancestor (BA) and CoS. “2^*^TSB + water (1^*^TSB)” is set as the control medium. Data: mean ± standard error; “2^*^TSB + CoS biofilm matrix”: 24 replicates from two CoS biofilms and two CoR isolates, others: 12 replicates from two CoR isolates.

Because cells and extracellular matrix are two major components of a microbial biofilm, we compared total cell numbers and their production of exopolysaccharide within biofilms. In monoculture biofilms, the rough type exhibited significantly lower cell numbers (Fig. 2A) and lower exopolysaccharide production (Fig. 2B) compared to the slimy morphotype, irrespective of the evolution treatment. One *S. degradans* evolved isolate SE4 was selected as representative and denoted as “SE”. Like the ancestor, SE was unable to form structured biofilm at the air-liquid interface (Fig. 1C, 2B, 2C).

To examine if evolutionary diversification affected bacterial interactions, we compared the dual-species biofilms formed by the two ancestral strains with biofilms formed by the evolved isolates of the two species. The CoR isolates exhibited significantly higher cell numbers in coculture with SE compared to CoS (CoR_SE vs CoS_SE) (Fig. 2D). In contrast, the MoR and MoS isolates did not show an increase in cell numbers in coculture with SE compared to the ancestor (MoR_SE, MoS_SE vs BA_SA) (Fig. 2D). In most cases, SE showed significantly higher cell numbers in coculture with *B. velezensis* evolved isolates compared to the dual ancestor combination (BA_SA), especially with CoR (CoR_SE) (Fig. 2D). Additionally, CoR_SE showed significantly higher exopolysaccharide production compared to BA_SA and MoR_SE (Fig. 2E), further demonstrating that both CoR and SE benefit in cocultures. MoS_SE also exhibited increased exopolysaccharide production, whereas MoS decreased cell numbers, and SE increased cell numbers compared to their respective ancestor, suggesting that SE is likely responsible for the increased production of exopolysaccharide.

To further dissect the effects of co-evolution history, we included additional pairwise cocultures between evolved and ancestral strains. When SE was paired with ancestral *B. velezensis* (BA_SE), no increase in *S. degradans* cell numbers was observed relative to the ancestral combination (BA_SA). When CoR was paired with ancestral *S. degradans* (CoR_SA), *S. degradans* cell numbers were significantly lower than in CoR_SE, suggesting that CoR confers greater benefits to SE than to SA. These results indicate that both the CoR and SE contribute to the improved performance of the coculture. In contrast, cocultures between CoS and SA or SE did not show significant differences in cell numbers or exopolysaccharide production (Fig. 2D, E). Based on these results, we conclude that the CoR isolates had a disadvantage in monoculture, whereas they benefitted in cocultures.

### Rough type exploits the biofilm former, but engages in a mutualistic interaction with the biofilm-beneficiary *S. degradans*

To validate the fitness advantage of CoR, we tested whether biofilm formation of CoR could be complemented by the extracellular matrix produced by the slimy *B. velezensis* isolates or the supernatants of the evolved *S. degradans* isolate. In line with this hypothesis, the CoR isolates significantly improved biofilm formation when supplemented with ancestor’s or slimy isolates' extracellular matrix, but not their own biofilm matrix (Fig. 2F). This result suggests that rough isolates act as exploiters in *B. velezensis* populations. In addition, the supernatant of evolved *S. degradans* (SE) also stimulated biofilm formation in rough isolates, whereas the ancestral supernatant (SA) had no such effect (Fig. 2F). The finding that both CoR and evolved *S. degradans* benefited from coculture conditions suggests a mutualistic interaction between both strains. Accordingly, rough isolates displayed an increased fitness in coculture evolved biofilms that was likely due to exploiting the extracellular substances that were released by its siblings or the co-existing species.

### Specific mutations drive phenotypic differentiation

Given the distinct adaptations observed under mono- and coculture conditions, we next investigated the genetic basis of such phenotypic differentiation. To this end, we sequenced the genomes of 69 *B. velezensis* evolved clones (16 volcano types, 25 slimy types, 26 rough types, and 2 wrinkle types) and 16 *S. degradans* evolved clones. In our analysis, we excluded mutations with low frequency (below 95%) to avoid sequencing errors, synonymous single nucleotide polymorphisms (SNPs) that do not impact gene function, and intergenic mutations with unknown effects on gene expression. This filtering allowed us to concentrate on significant, high-frequency, non-synonymous mutations within coding regions. Additionally, the genomes of the two ancestors were also re-sequenced to screen for mutations that emerged before the evolution experiment. Comparing the genome of ancestral *B. velezensis* and *S. degradans* to the genome sequence that is available at NCBI revealed 17 and seven mutations, respectively (Supplementary Dataset S2), which were excluded from further analysis.

**Figure 3:**
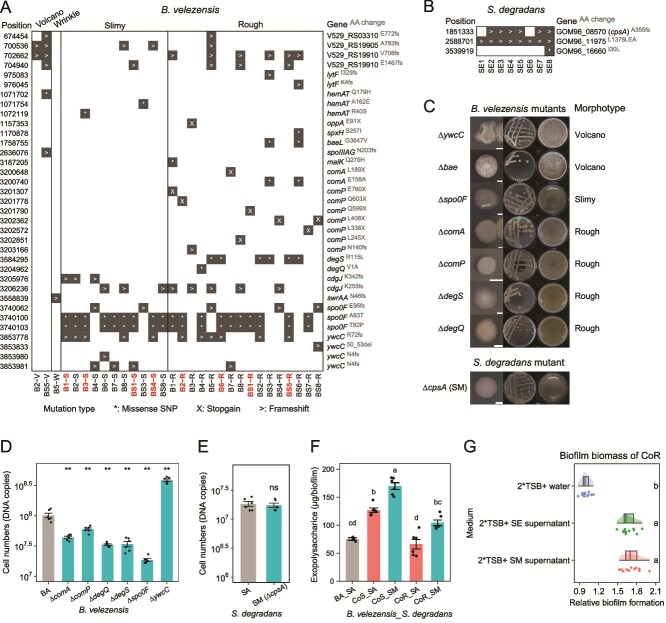
Common mutations identified in evolved clones from 18^th^ transfer and the representative mutants. (**A**) *B. velezensis* SQR9 mutations. Isolates labeled in red were selected as representative clones for CoR, CoS, MoR, and MoS. V abbreviates for volcano, W abbreviates for wrinkle, S abbreviates for slimy, R abbreviates for rough. The left-side labels indicate chromosomal positions of mutation, and the right-side labels indicate the corresponding genes and amino acid changes. (**B**) *S. degradans* XL272 mutations. SE abbreviates for evolved *S. degradans*. For both (A) & (B), “n” indicates the population. “^*^” refers to missence single nucleotide polymorphism, “>” refers to frameshift mutation, and “X” refers to stop-gain mutation. All the isolates shown above were isolated from the 18^th^ transfer. Isolates from the 9^th^ transfer were shown in Fig. S4A and S4B. (**C**) Colony and pellicle morphotype of mutants. The left column shows an enlarged view of a colony, the middle column shows the entire plate of streaked colonies, and the right column shows the pellicle phenotype. Colonies with different morphotypes were streaked on TSB agar plates and cultivated at 30°C. Pellicles were cultivated in TSB liquid medium at 30°C, and photographs were taken 48-hour post-incubation. Scale bar represents 0.2 cm, Petri dish diameter is 9 cm, well diameter is 1.5 cm. (**D**) Cell numbers quantification of *B. velezensis* monoculture biofilms by qPCR. n = 6. “^*^^*^” indicates significant difference compared to “BA” (t test, *P* < .01). (**E**) Cell numbers quantification of *S. degradans* monoculture biofilms by qPCR. n = 6. “ns” indicates no significant difference compared to “SA” (t test, *P* < .01). (**F**) Exopolysaccharide quantification of biofilms. BA_SA: three replicates, others: six replicates. Different letters indicate significant differences based on ANOVA, Tukey’s posthoc test (*P* < .05). Data shows the mean ± standard error. (G) Relative biofilm of CoR isolates in media supplemented with supernatant from *S. degradans* isolates, compared to that in TSB medium. Biofilm biomass was quantified by measuring the OD_600_ of vortexed biofilm. “2^*^TSB + water (1^*^TSB)” was set as the control; n = 6 for control, n = 12 for each test condition (from two CoR isolates). Data represent mean ± standard error. Different letters indicate significant differences based on ANOVA, Tukey’s posthoc test (*P* < .05).

Focusing on mutations that were present in at least two clones (Fig. 3A and S4A), we observed that in *B. velezensis*, the most frequently mutated genes were *ywcC*, which encodes a regulator controlling the expression of the *slrA* gene, and *spo0F*, which encodes a phosphotransferase of the sporulation initiation phosphorelay. These two genes harbored missense SNPs or frameshift mutations at different positions. SlrA, together with SlrR, forms a regulatory complex (SlrA/SlrR) that stimulates biofilm formation by antagonizing the global negative regulator of biofilm development, SinR. In *Bacillus subtilis*, the *ywcC* mutation enhances SlrA/SlrR activity, thereby promoting biofilm formation [40]. To verify their roles, we constructed knockout mutants of *ywcC* and *spo0F* in the ancestor background. The ∆*spo0F* mutant displayed a slimy colony phenotype with reduced biofilm formation, whereas the ∆*ywcC* mutant, although morphologically similar to the ancestor, showed enhanced biofilm formation (Fig. 3C, D). These findings suggest that the slimy colony morphotype is likely caused by the *spo0F* mutation, whereas the enhanced biofilm formation of slimy type is likely due to the *ywcC* mutation.

Rough morphotype carried mutations in either the *comA*/*comP* or *degS*/*degQ*. The ComP-ComA two-component signal transduction system regulates quorum sensing and competence development in *B. subtilis* species [41, 42]. The sensor kinase DegS, together with its response regulator DegU, coordinate multicellular behavior including biofilm formation, genetic competence, motility, degradative enzyme production, and poly-γ-glutamic acid production [43]. DegQ protein enhances the phosphate transfer from DegS to DegU, deletion of the *degQ* gene impairs complex colony architecture and biofilm formation [44]. Knockout mutants of these four genes all displayed a rough colony phenotype and reduced biofilm formation (Fig. 3C, D). The two wrinkled clones shared frameshift mutation in the *swrAA* gene that encodes for the swarming motility protein [45].

In *S. degradans*, only three parallel mutations were identified (Fig. 3B). One of which was shared in all clones: a frameshift insertion in the GOM96_11975 gene encoding a histidine kinase of unknown function. This mutation was likely acquired in the overnight culture of the ancestor that was used to initiate the evolution experiment. Most clones shared an identical frameshift insertion at the high C region in the GOM96_08570 gene that encodes a capsular polysaccharide (CPS) biosynthesis protein, we designated *cpsA*. The production of CPS was reported to interfere with biofilm formation across various bacterial species [46–48]. Given that biofilms of evolved cocultures became slimy, we hypothesized that this gene could be potentially linked to polysaccharide production. To test this, we constructed a complete knockout strain ∆*cpsA* (SM) in the ancestor background (Fig. 3C). The mutant exhibited a round colony edge resembling that of the evolved *S. degradans* clone (SE) (Fig. 1C), and failed to form structured biofilm at the air-liquid interface. The total cell numbers in monoculture biofilms did not significantly differ between SA and ∆*cpsA* (Fig. 3E)*.* The dual-species biofilm containing the ∆*cpsA* mutant produced more exopolysaccharides compared to coculture with the *S. degradans* ancestor (Fig. 3F). This effect was not due to an increase in biomass, as quantified by OD_600_ (Fig. S4C), indicating that the disruption of the *cpsA* gene is likely responsible for the higher production level of exopolysaccharide. Moreover, the ∆*cpsA* supernatant promoted biofilm formation of CoR isolates, similar to the evolved variant (SE) (Fig. 3G), further supporting its functional resemblance to SE.

Taken together, our genomic analysis reveals that specific mutations drive phenotypic differentiation. In *B. velezensis*, *spo0F* mutations were linked to a slimy colony morphology, whereas mutations in *comA*/*comP* and *degS*/*degQ* were associated with rough morphologies. Additionally, *ywcC* mutations were linked to enhanced biofilm formation. Finally, in *S. degradans*, *cpsA* mutations increased exopolysaccharide production in dual-species biofilms.

### Biofilm beneficiary changes the mutational dynamics of the biofilm former

To understand how these mutations emerged and spread over time under different ecological conditions, we conducted longitudinal population sequencing of all lineages at the first and every third transfer. We filtered out synonymous substitutions, mutations that did not reach a cumulative frequency of 5% within a lineage, and those were present already in the ancestral genome. The filtered mutations covered a broad range of genetic changes including missense and nonsense substitutions, InDels (insertions and deletions) in coding regions, as well as changes in noncoding DNA such as intergenic regions, tRNA, and rRNA. Based on their emergence, disappearance, and persistence, mutations were categorized into three groups: (i) fixed mutations, (ii) transient mutations, and (iii) fluctuating mutations. Fixed mutations are genetic changes that achieved a 100% frequency across all individuals in a population by the end of this study. Transient mutations only appear temporarily at a single transfer and disappear after that. These mutations are likely deleterious and are eliminated by selection, or they are neutral and eventually lost from the population due to genetic drift [49]. Fluctuating mutations are those vary in frequency over time, but not disappear completely. These mutations might provide a moderate selective advantage.

**Figure 4:**
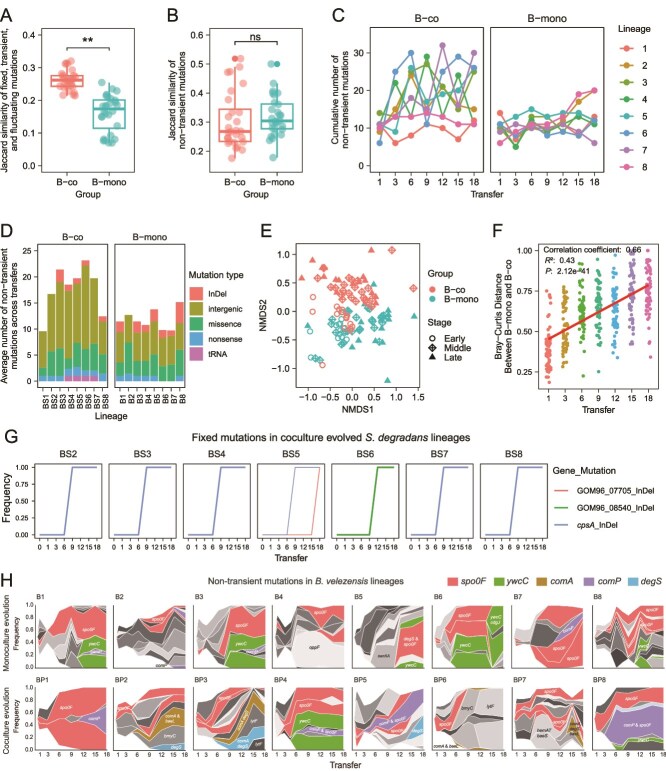
Mutational dynamics of populations. (**A, B**) Degree of parallelism within each group estimated by Jaccard index. The Jaccard index describes the likelihood that the same gene is mutated in two independent lineages and ranges from 0 (no mutated genes in the two lineages are shared, G1∩G2 = 0) to 1 (the two lineages have exactly the same set of mutated genes, G1 = G2). Dots indicate the J value of paired-wise comparison between lineages. (**A**) Jaccard index calculated using fixed, transient, and fluctuating mutations. Asterisks indicate significant differences between coculture evolved and monoevolved *B. velezensis* (t test, ^*^^*^P < .01). (**B**) Jaccard index calculated using fixed and fluctuating mutations. (**C**) Cumulative number of non-transient mutations detected in each lineage over time. (**D**) Distribution of mutation type of each lineage. Average number of non-transient mutations across transfers are shown. (**E**) Non-metric multidimensional scaling (NMDS) ordinations of coculture-evolved and monoevolved *B. velezensis* populations based on a bray–Curtis dissimilarity matrix. “Early” represents 1^st^ and 3^rd^ transfer, “middle” represents 6^th^, 9^th^, and 12^th^ transfer, “late” represents 15^th^ and 18^th^ transfer. (**F**) Bray-Curtis distance between the mutations of monoevolved and coculture-evolved *B. velezensis* lineages over time. Distance was calculated using non-transient mutations. (**G**) Mutational trajectory of fixed mutations in coculture evolved *S. degradans* populations. Colour shows different gene. No fixed mutation was identified in BS1, thus not shown. (**H**) Genealogy and genotype frequencies of *B. velezensis* over time. Each genotype comprises one or more co-occurring non-transient mutations. The colours represent different genotypes, with the vertical area indicating genotype frequency as determined by Lolipop. When a mutation arises in the background of another mutation, generating a new genotype, the new colour arises in the middle of the existing genotype. Genotypes carrying key mutations are highlighted in certain colours and annotated with the corresponding genes, whereas all other genotypes are shown in grey.

We compared the mutational dynamics of *B. velezensis* that evolved under mono- and coculture conditions. First, we assessed the parallelism of the mutations by calculating the Jaccard similarity coefficient (*J*). The higher the value *J*, the more similar two lineages are with regards to their mutations. *J* values of monoevolved *B. velezensis* lineages were significantly lower than those of coevolved lineages (t test, *P* < .01) (Fig. 4A), indicating a lower degree of genetic parallelism in monoevolved lineages. Excluding transient mutations from the analysis increased *J* values for both conditions, yet removed the significant difference in *J* values between both types of lineages (t test, *P* = .52, Fig. 4B). This observation suggests that the previously observed low degree of parallelism in monoevolved populations was likely due to a larger number of transient mutations in these lineages.

To avoid potential bias caused by transient mutations, we also filtered them out in subsequent analyses (Fig. 4C-F). Most of the remaining mutations occurred in intergenic regions (Fig. 4D). We applied a linear mixed-effects model (LME), fitted using restricted maximum likelihood to analyze the differences in the cumulative number of non-transient mutations under two evolutionary conditions: *B. velezensis* in monoculture (B-mono) or coculture (B-co). This model included evolutionary condition, transfer, and their interaction as fixed effects, with lineage as a random intercept to account for variations among independent evolutionary lines. A type-III analysis of variance (ANOVA) was performed on the fitted model to evaluate the significance of main effects and their interaction. Post hoc pairwise comparisons between groups at each transfer point were conducted based on estimated marginal means, with degrees of freedom approximated by the Kenward-Roger method for the associated t tests. At transfer 3, 6, 9, 12, 18, coculture-evolved *B. velezensis* populations accumulated significantly more non-transient mutations than monoevolved *B. velezensis* populations (LME, Type III ANOVA, post hoc pairwise comparisons, all *P* < .05). For example, at transfer 6, B-co populations had, on average, eight more mutations than B-mono populations (Estimate = 8.00, t(91) = 3.20, *P* = .0019), whereas no significant differences were observed at transfer 1 (Estimate = 0.13, t(91) = 0.05, *P* = .96) and transfer 15 (Estimate = 4.50, t(91) = 1.80, *P* = .075). When considering all transfers together, the overall effect of cultivating *B. velezensis* in mono- versus coculture conditions was not statistically significant (Estimate = −0.13, t(91) = −0.05, *P* = .96), suggesting that the difference of mutation accumulation observed in later transfers was likely offset by the lack of differences at certain transfers (i.e. transfers 1 and 15). The factor “lineage” contributed moderate variability (Variance = 2.07, SD = 1.44), suggesting that although different lineages influence mutation counts, they do not dominate the overall pattern.

Nonmetric multidimensional scaling (NMDS) analysis based on Bray–Curtis dissimilarity revealed that in early evolutionary stages (1^st^ and 3^rd^ transfer), mutations in monoevolved populations were largely overlapped with mutations on coculture-evolved populations (Fig. 4E). However, the mutations grouped separately during the middle and late stages of evolution (6^th^ to 18^th^ transfer). Permutational multivariate ANOVA (PERMANOVA) based on Bray-Curtis dissimilarities showed that the mutation compositions between the two evolution groups differed significantly (*R*^2^ = 0.10, *F* = 12.25, *P* < .001). Accordingly, a linear model showed that the Bray-Curtis distance increased significantly over time between monoevolved and coculture evolved *B. velezensis* populations (*F*(6,441) = 56.35, *R*^2^ = 0.43, *P* < .001) (Fig. 4F), indicating the dissimilarity in the spectrum of mutations between the two evolutionary groups increased over time.

To further explore the divergence between the two evolution groups, we performed an analysis in which we specifically focused on fixed mutations. In monoevolved *B. velezensis* populations, mutations in the *spo0F*, *ywcC*, and *cdgJ* genes were fixed in certain lineages (Fig. S5A). Parallel mutations in the intergenic region between the *aroA* gene and the V529_29720 gene were identified in both monoevolved and coculture evolved *B. velezensis* populations, indicating strong selection, although their impact remains to be determined. In coculture evolved *B. velezensis* populations, there was a variety of fixed mutations across lineages, affecting genes associated with colony morphotype, including *comA*, *degS*, *spo0F*, and *ywcC* (Fig. S5B). For *S. degradans*, insertions in the *cpsA* gene were consistently fixed across six populations (Fig. 4G).

Fluctuating mutations, although not fixed by the end of the experiment, also contributed to within-population diversity. Including both fluctuating and fixed mutations, we inferred the genealogy of genotypes within each population/community using the lollipop software package [39]. We focused this analysis on mutations that occurred in coding regions, thus excluding mutations found in intergenic regions. The linked genotypes and ancestry were identified based on allele frequency data and displayed as Muller plots (Fig. 4H). Each genotype consists of one or more co-occurring non-transient mutations. The resulting data show the breadth of genotype frequency with colours representing the presence of *spo0F*, *ywcC*, *comA*, *comP*, *degS*, and *baeL* mutations within genotypes. Regardless of the evolution treatment, *spo0F* mutations were detected in all lineages and subsequently rose in frequency, reflecting adaptation to the TSB medium. The biofilm-enhancing *ywcC* mutations were present in five of monoevolved lineages, which is consistent with the increased population size in monoculture evolution (Fig. 1C). Mutations causing rough morphotype (*comA*, *comP*, *degS*) were observed in all coculture-evolved lineages, but only in five of monoevolved lineages. Mutations in *comA* and *baeL* were exclusively found in three coculture-evolved lineages and always cooccurred within the same genotype.

### Coevolution with a *S. degradans* variant or a Δ*cpsA* mutant accelerates the fixation of the rough type in *B. velezensis*

**Figure 5:**
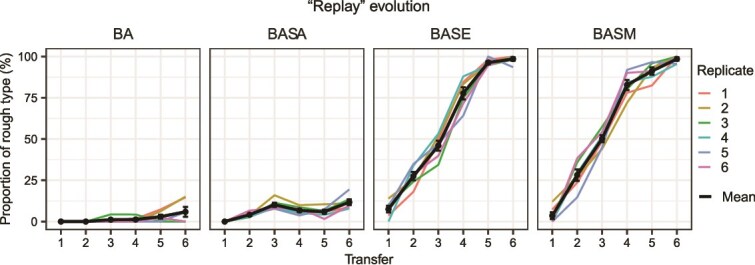
Proportion of rough type in “replay” evolution treatments. Coculture evolution with the evolved variant of *S. degradans* or the Δ*cpsA* mutant accelerates the fixation of the rough type in the *B. velezensis* population. “BA” represents the *B. velezensis* ancestor evolving in isolation, “BASA” represents the *B. velezensis* ancestor evolving alongside the *S. degradans* ancestor, “BASE” represents the *B. velezensis* ancestor evolving with the evolved isolate of *S. degradans*, and “BASM” represents the *B. velezensis* ancestor evolving with the *S. degradans* Δ*cpsA* mutant. The data points show the mean proportion of the rough type across six replicates, along with their standard errors, calculated over six transfers.

The observed rough type of *B. velezensis* might reflect a specific adaptive response to the biofilm cultivation environment, the biotic interaction with *S. degradans*, or be due to a combination of both. The relative frequency of the rough type began to increase in the coculture treatment starting from the 6^th^ transfer. At the same time, mutations in the *cpsA* gene of *S. degradans* emerged. This correlation could be due to a causal relationship. To assess whether the competitive advantage of the rough type in *B. velezensis* over the slimy type that evolved in coculture was a direct response to the mutations in the *cpsA* gene that emerged in *S. degradans*, we “replayed” the corresponding situation in the evolution experiment. For this, we not only repeated the monoulture and coculture treatment of the original experiment, but also included two additional coculture treatments, in which the ancestral strain of *B. velezensis* was serially propagated together with either the evolved clone of *S. degradans* or the Δ*cpsA* mutant. The proportion of rough types that emerged in these cultures until the 6^th^ transfer was recorded. When evolved together with either the evolved clone of *S. degradans* or the Δ*cpsA* mutant (Fig. 5), the rough type rapidly emerged in *B. velezensis* and became fixed in the population. These findings indicate that a loss-of-function mutation in the *cpsA* gene was sufficient for the fixation of the rough type in the *B. velezensis* populations.

In total, 21 rough isolates were obtained from the 6^th^ transfer of the “replay” experiment. As observed previously, the isolates rough type carried mutations in at least one of the following genes: *comA*, *comP*, *degS*, and *degQ* (Table 1).

**Table 1:** Mutations detected in rough isolates from "replay" evolution treatments.

Isolate	Replay evolution treatment	*comA*	*comP*	*degS*
1	BA	542 C > A missence		38 - < C Frameshift
2		542 C > A missence		
3			673 C > T missence	
4	BASA	542 C > A missence		
5		542 C > A missence		
6		542 C > A missence		
7		542 C > A missence		
8			673 C > T missence	
9		542 C > A missence		
10	BASE	542 C > A missence		
11		542 C > A missence		
12		542 C > A missence		
13		542 C > A missence		
14		542 C > A missence		
15		542 C > A missence		
16	BASM		1162 A < G missence	
17			673 C > T missence	
18		542 C > A missence		
19		542 C > A missence		
20		542 C > A missence		
21		542 C > A missence		

The numbers under the gene names indicate the positions of detected mutations, followed by detailed information on the type of mutation. Full sequences of the *comA*, *comP*, *degS*, and *degQ* genes from rough isolates obtained after the sixth transfer of the "replay" evolution treatments were determined using Sanger sequencing. No mutations were detected in the *degQ* gene; therefore, this gene is not included in the table.

## Discussion

In their natural environment, microorganisms do not evolve in isolation but coexist and interact with many other species that are part of the same community. Interspecies interactions have been previously shown to affect adaptive evolution of the strains involved [29, 50–54]. Here we addressed the following main question: How does a biofilm beneficiary affect the evolution of a biofilm former? Our study shows that the biofilm-beneficiary *S. degradans* did not evolve enhanced biofilm formation, but increased its dependence on *B. velezensis*. Two types of colony morphologies evolved in the biofilm former: the biofilm-enhanced slimy type, which showed highest fitness under monoculture conditions, and the biofilm-weakened rough type, which exploits the extracellular matrix from the biofilm community. Coevolution with *S. degradans* increased the fitness of the rough type within the *B. velezensis* population. Specifically, mutations in the *cpsA* gene of *S. degradans* increased exopolysaccharide production in the dual-species biofilm, providing the rough variant with a fitness advantage over the slimy variant in the *B. velezensis* population.

When evolving under monoculture conditions, *B. velezensis* evolved morphotypes that resemble those that have been previously reported in other *Bacillus* species, particularly in terms of extracellular matrix production and sporulation. First, variation in extracellular matrix production has been previously shown to be critical during biofilm evolution [9, 11–13, 20]. Under standard laboratory culturing conditions, uniformly enhanced biofilm formation is not favored. For instance, in *B. subtilis*, most cells diversified into morphotypes that produce less biofilm matrix than their ancestor, although some overproducing variants were also present [55]. In our study, the slimy and rough types were associated with varying levels of extracellular matrix production. The increased biofilm formation of the slimy type was likely due to mutations in *ywcC*. The *ywcC* locus has been identified as a mutational hotspot when *B. subtilis* evolves an enhanced biofilm formation or root colonization [56, 57]. Further mutations in *comA*, *comP*, *degS*, or *degQ* genes might counteract the effect of mutations in *ywcC* and led to impaired biofilm formation of the rough isolates [42, 44]. Similar to the smooth variants of *B. subtilis* [10], the rough variants identified in this study took advantage of the biofilm matrix components that have been secreted by other morphotypes. Moreover, the adaptive evolution of variants that differ in their production level of polysaccharides have been previously reported to emerge in biofilms of *Burkholderia cenocepacia* [6, 58, 59] and *Pseudomonas* species [60–62]. Second, several evolved isolates of *B. velezensis* carried mutations in the *spo0F* gene, which hindered spore formation in the corresponding mutants. This observation is strongly reminiscent to previously reported experiments in which *B. thuringiensis* formed biofilms on beads [63]. Sporulation is an essential trait for surviving harsh conditions in the soil, which is the preferred habitat of the focal species. However, spore formation was not selectively favored in the experimental conditions of this study. Also, other studies have reported a loss of sporulation during laboratory-based cultivation in rich media [64, 65]. These observations are consistent with the interpretation that phenotypic traits are lost when selection for that phenotype is relaxed or eliminated [66].

In coculture evolution, similar *B. velezensis* morphotypes appeared; however, the rough type emerged faster and became more prevalent than in monoevolution. This observation is reminiscent of another biofilm evolution study, of which the wrinkled variant of *Xanthomonas retoflexus* emerged in both in monoevolution and dual-species biofilm evolution. In that research, the selective pressure applied by *Paenibacillus amylolyticus* increased the frequency of the wrinkled variant and reinforced mutualism between both species [22]. Selection on the rough variants of *B. velezensis* population were associated with a frameshift mutation in the *cpsA* gene of *S. degradans*, encoding CPS biosynthesis protein. This mutation resulted in increased exopolysaccharide production by *S. degradans*, which could serve as shared resources for the consortium that formed the dual-species biofilm. In *Pasteurella multocida* and *Vibrio vulnificus*, mutants lacking CPS were found to form significantly thicker biofilms than their wild type counterparts [46, 47]. In addition, deletion of *cps* from the genome of *Klebsiella variicola* impacting traits such as aggregation, virulence, and morphotype differentiation when being cultivated in spatially structured environments [67–69]. Evidence from our “replay” experiment indicated that the ∆*cpsA* mutant accelerated the fixation of the rough variant of *B. velezensis*. Comparative secretome or matrix composition analyses of ancestral versus ∆*cpsA* strains, as well as matrix complementation assays, could reveal whether the qualitative properties of shared public goods influence the selection of exploiters. Similarly, coevolution experiments with *Pseudomonas putida* KT2440 and *Acinetobacter johnsonii* C6 revealed that the rough variant of *P. putida* only outcompeted its ancestor in the presence of *A. johnsonii* and in spatially structured environment, but not when growing under monoculture conditions [24]. In that research, mutations in the *wapH* gene, which affects lipopolysaccharide biosynthesis, were identified to be causally involved in the generation of the rough variant of *P. putida*. Likewise, lipopolysaccharide mutations of *Pseudomonas aeruginosa* occurred as a direct consequence of selection imposed by *Staphylococcus aureus* [28]. In addition, mutations in the *baeL* gene of *B. velezensis* emerged in our coculture evolution, which is involved in bacillaene synthesis. When coevolving with *Pseudomonas fluorescens* on tomato roots, *B. subtilis* displayed mutations in the *pksR* gene, which is also involved in synthesizing bacillaene [56]. Bacillaene has been reported to exhibit bacteriostatic activity against *P. chlororaphis* [70]. The mutation in the *baeL* gene could therefore be a strategy to minimize inhibitory effects on *S. degradans*.

Despite the increased prevalence of exploitative “rough” variants in coculture, the final biofilm biomass was lower than in monocultures. This suggests that the evolutionary success of these variants does not necessarily translate to higher community-level productivity. Instead, it reflects a shift in social structure—where certain individuals reduce their own investment in public goods but benefiting from others. Such social diversification may help stabilize coexistence rather than maximize total biomass. Future work should evaluate how evolutionary trajectories under coculture conditions affect the overall functionality of microbial consortia.

In summary, our study reports that a biofilm beneficiary can increase the fitness of an extracellular matrix-exploiting variant that emerged in the population of a biofilm former. Conceptually, this finding is in line with the Black Queen Hypothesis (i.e. the evolution of dependency through trait loss) [71]. The experimental framework developed here provides a useful model for exploring how interspecies interactions shape social evolution. Our findings underscore the importance of ecological context in driving microbial diversification.

## Supplementary Material

Supplementary_figures_wraf160

Dataset_S1_primers_wraf160

Dataset_S2_Genome_resequencing_isolates_wraf160

Dataset_S3_Population_sequencing_mutations_wraf160

Dataset_S4_Main_figure_data_2025_wraf160

Dataset_S5_Supplementary_figure_data_wraf160

## Data Availability

Data used in the main figures were provided in Supplementary Dataset S4. Data used in the supplementary figures were provided in Supplementary Dataset S5. Other datasets have been mentioned in Materials and Methods. Codes for analyzing the population mutations are detailed in https://github.com/XinliSunNJAU/Bacillus-Pseudomonas-biofilm-evolution.git. Raw sequencing data have been deposited into CNGB Sequence Archive (CNSA) of China National GeneBank DataBase (CNGBdb) with accession number CNP0003899.
