# Supplementary material for: Presence of a biofilm beneficiary alters the evolutionary trajectory of a biofilm former
Source: ISME J. 2025 Jul 31;19(1):wraf160. doi: 10.1093/ismejo/wraf160 (PMC12393148; doi:10.1093/ismejo/wraf160)
Supplement: Supplementary_figures_wraf160 [file supplementary_figures_wraf160.pdf]

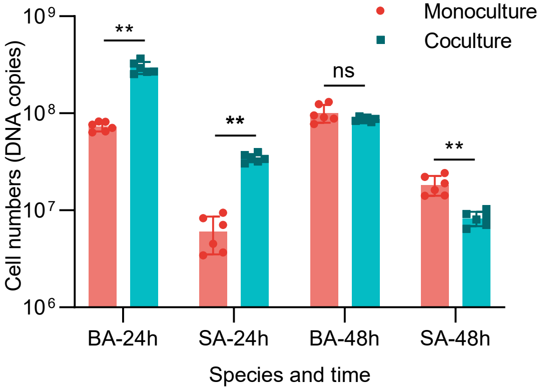

Fig. S1 Cell numbers quantification in pellicle. Dual-species pellicle containing ancestral isolates of 24h and 48h. “BA” represents *B. velezensis* ancestor, “SA” represents *S. degradans* ancestor. “**” indicates significant differences (P < 0.01) based on t test.

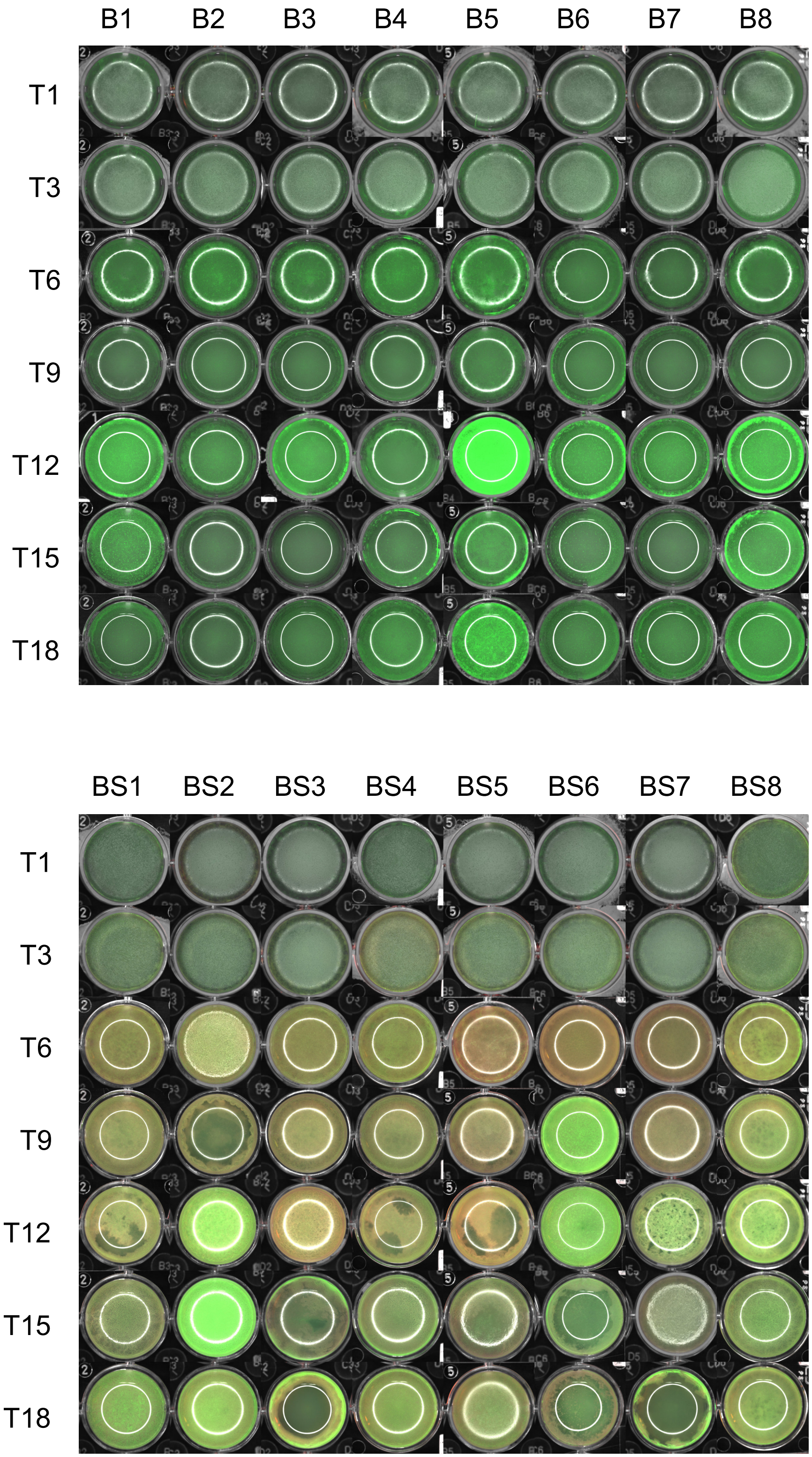

Fig. S2 Phenotype of evolving pellicle populations. (A) Mono-evolution. **(B)** Co-evolution. *B. velezensis* were labeled green, *S. degradans* were labelled red, the overlay were yellow or orange depending on the proportion of the two species. The bright ring reflects the moisture level of the pellicle—the higher the moisture content, the more pronounced the reflective halo.

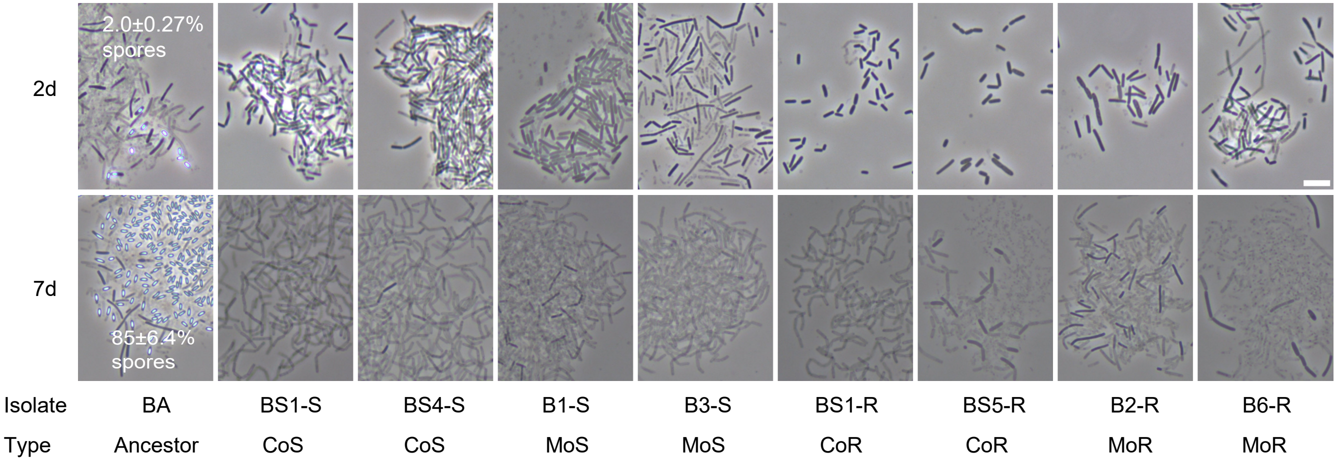

Fig S3. Disability of sporulation by evolved *B. velezensis* morphotypes. Representative images of sporulation in biofilms. Samples were collected 2 days and 7 days post incubation, and votexed rigorously. Scale bar represents 5 μm. No spores were observed except the ancestor.

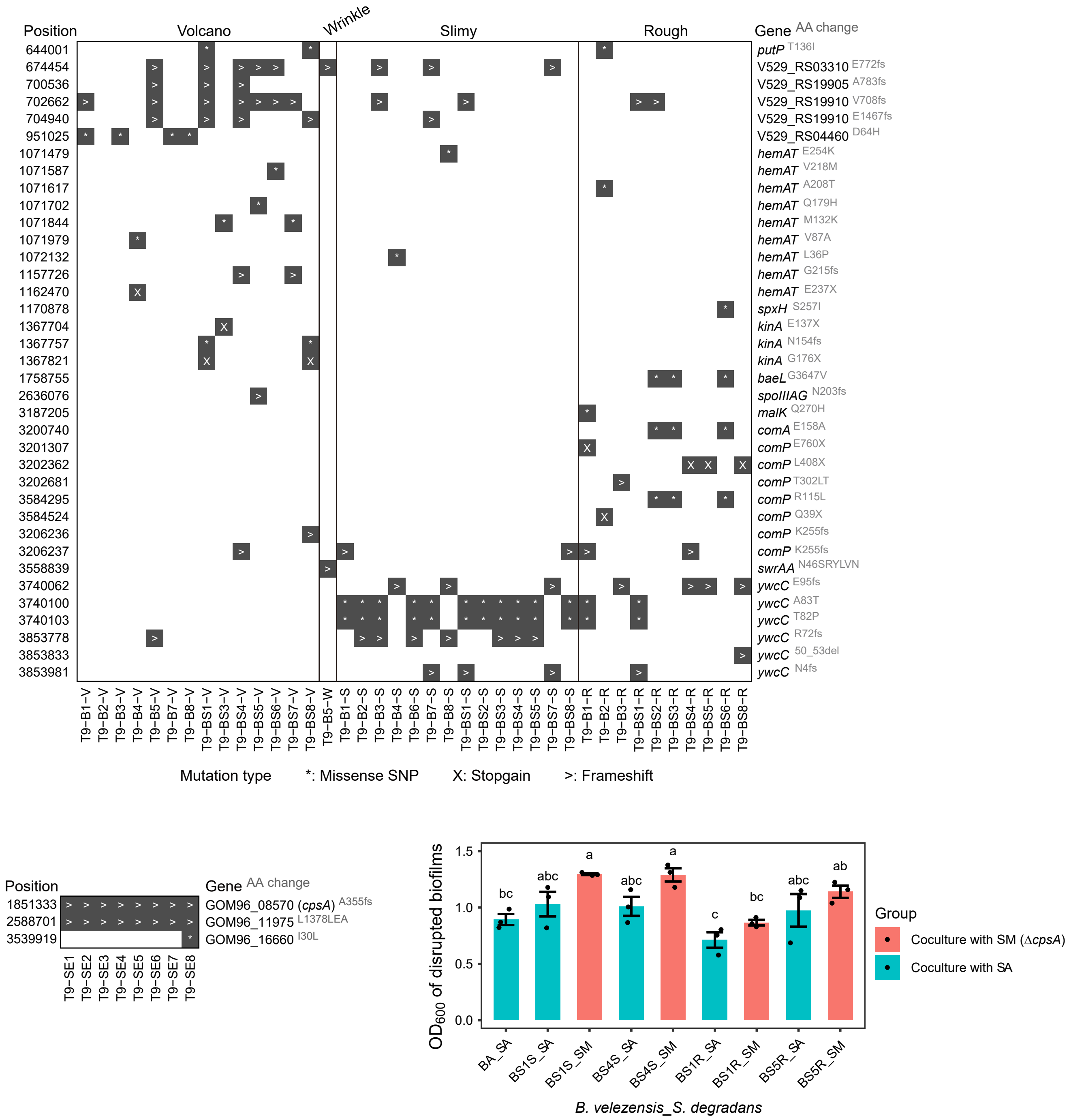

Fig. S4 Mutations identified in evolved clones from 9th transfer. (A) *B. velezensis* SQR9 mutations. The left-side labels indicate chromosomal positions of mutation, and the right-side labels indicate the corresponding genes and amino acid changes. V abbreviates for volcano, W abbreviates for wrinkle, S abbreviates for slimy, R abbreviates for rough. **(B)** *S. degradans* XL272 mutations. SE abbreviates for evolved *S. degradans*. For both (A) & (B), "n" indicated the population. "*" refers to missence single nucleotide polymorphism, ">" refers to frameshift mutation, "X" refers to stopgain mutation. All the isolates shown above were isolated from the 9th transfer. **(C)** Biofilm quantification by measuring the OD_{600} of disrupted biofilms. BA_SA: 3 replicates, others: 6 replicates. Different letters indicate significant differences based on ANOVA, Tukey's posthoc test (P < 0.05). Data shows the mean ± standard error.

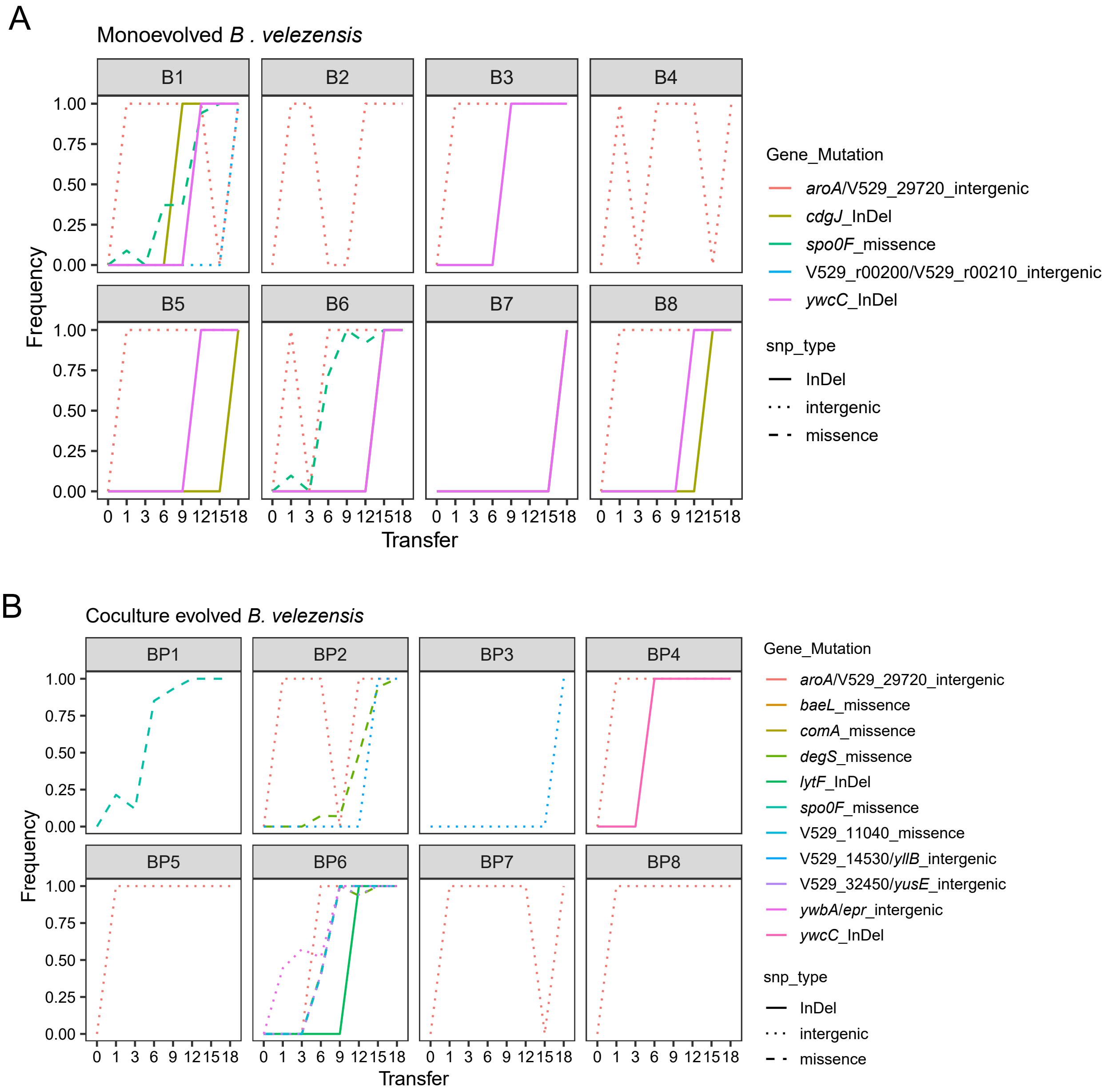

Fig. S5 Mutational trajectory of fixed mutations. (A) Mono-evolved *B. velezensis* population. (B) Coculture evolved *B. velezensis* population.
